# Editorial Lp(a) – the underestimated cardiovascular risk factor

**DOI:** 10.1007/s11789-017-0091-2

**Published:** 2017-02-24

**Authors:** Klaus-Peter Mellwig

**Affiliations:** Klinik für Kardiologie, Herz- und Diabeteszentrum NRW, Ruhr-Universität Bochum, Georgstr. 11, 32545 Bad Oeynhausen, Germany

Despite optimized therapeutic approaches, coronary heart disease with its secondary diseases, such as myocardial infarction and chronic heart failure, as well as embolic stroke are the main causes of morbidity and mortality. They result from inflammatory processes induced by lipoproteins such as oxidized low-density lipoprotein (LDL), VLDL and lipoprotein(a) (Lp(a)).

Fifty years after the first publication by Kare Berg from Oslo, Lp(a) has meanwhile been accepted as an individual risk factor. However, in spite of the increasing scientific interest in Lp(a), biological function and biosynthetic and catabolic processes of this molecule are still widely unknown. Furthermore, the exact laboratory determination of Lp(a) plasma concentration and its different isoforms is still challenging.

The individual Lp(a) serum concentration is genetically determined and remains relatively constant thoughout life. Some ethnic groups in Arabic and African countries show very high Lp(a) plasma concentrations.

In the meantime, several registries have indicated that a high Lp(a) plasma concentration is associated with an increased incidence of coronary heart disease, aortic valve stenosis and heart failure. However, there is still no systematic analysis of the population in order to assess their cardiovascular risk depending on Lp(a).

For the first time, the European Society of Cardiology has now included Lp(a) determination in the extended screening for the assessment of the cardiovascular risk.

In Germany, there are still no effective drugs to lower Lp(a) plasma concentration. Neither are the recently approved PCSK9 inhibitors expected to significantly improve therapeutic options. Thus, lipid apheresis has to be regarded as the only effective therapeutic option to decrease cardiac events.

Against this background, the contributions to the present supplement are intended to focus on the clinical aspects of Lp(a) and to emphasize the importance of lipid apheresis.

